# Exploring the Intersection of Atypical Hemolytic Uremic Syndrome and Substance Use: A Comprehensive Narrative Review

**DOI:** 10.7759/cureus.71019

**Published:** 2024-10-07

**Authors:** Abrisham Akbariansaravi, Anushka Dekhne, Archi Dhamelia, Mario Mekhail

**Affiliations:** 1 Internal Medicine, American University of Antigua, Antigua, ATG; 2 Internal Medicine, MGM (Mahatma Gandhi Mission) Medical College, Navi Mumbai, IND; 3 Internal Medicine, Long Island Community Hospital, Patchogue, USA; 4 Intensive Care Unit, Ain Shams University, Cairo, EGY

**Keywords:** atypical hemolytic uremic syndrome, complement activation, diagnosis, genetic predisposition, management, pathophysiology, substance use

## Abstract

Hemolytic uremic syndrome (HUS) is a thrombotic microangiopathy characterized by hemolytic anemia, renal failure, and thrombocytopenia. While the typical form of HUS is often associated with Shiga toxin-producing *Escherichia coli* (STEC) infections, atypical hemolytic uremic syndrome (aHUS) is caused by uncontrolled complement system activation, leading to endothelial damage, microthrombi formation, and other complications. Although aHUS is commonly linked to genetic mutations and infections, emerging evidence suggests that certain substances, particularly illicit drugs like heroin, cocaine, and ecstasy, can also trigger this condition, adding complexity to its diagnosis and management.

This narrative review examines the mechanisms by which substance use can lead to aHUS, discusses its clinical presentation, and highlights the diagnostic challenges in distinguishing it from other thrombotic microangiopathies, such as thrombotic thrombocytopenic purpura (TTP) and STEC-HUS. A thorough literature search identified relevant case reports, case series, and observational studies, underscoring the need for genetic testing and complement assays to confirm aHUS in substance users. The review also explores the role of complement inhibitors, such as eculizumab and ravulizumab, which target the underlying pathophysiology and have shown promise in improving patient outcomes. However, the management of substance-induced aHUS remains challenging due to limited data, varying clinical presentations, and the need to optimize treatment protocols. Early recognition and tailored therapy are crucial for effective management. Further research is needed to refine diagnostic criteria, develop new therapeutic approaches, and improve care for patients with this under-recognized condition.

## Introduction and background

Atypical hemolytic uremic syndrome (aHUS) is a rare but life-threatening form of thrombotic microangiopathy (TMA) characterized by microangiopathic hemolytic anemia, thrombocytopenia, and acute kidney injury. Unlike typical hemolytic uremic syndrome (HUS), which is commonly associated with Shiga toxin-producing *Escherichia coli* (STEC) infections, aHUS is primarily driven by dysregulation of the complement system, a critical component of the innate immune response that defends against pathogens and maintains immune homeostasis [1]. When this regulatory system fails, it leads to uncontrolled complement activation, resulting in endothelial cell injury, platelet activation, and the formation of microthrombi in the microvasculature, which are defining features of both HUS and aHUS, with aHUS being primarily driven by complement dysregulation [1].

The development of aHUS is influenced by both genetic and environmental factors. Genetic mutations are implicated in 40-60% of aHUS cases, particularly in genes encoding complement regulatory proteins such as CFH (complement factor H), CFI (complement factor I), CD46 (membrane cofactor protein, MCP), C3, and CFB (complement factor B) [2]. These mutations lead to either a loss of function in complement regulators or a gain of function in complement activators, causing unregulated complement activation and endothelial damage. Mutations in CFH are the most common, accounting for about 25% of cases, while CD46 (MCP) mutations are present in 10-15% of patients, illustrating the genetic diversity contributing to aHUS [2].

Acquired factors, including autoantibodies against complement factor H (anti-CFH antibodies), play a crucial role in approximately 10% of aHUS cases [2]. These autoantibodies disrupt CFH’s ability to regulate complement activation on cell surfaces, further promoting endothelial injury. Their presence requires tailored therapeutic approaches compared to cases solely due to genetic mutations, highlighting the need for individualized treatment strategies [2].

Though genetic predispositions are fundamental, environmental or acquired triggers often precipitate aHUS in susceptible individuals. These triggers include infections, pregnancy, malignancies, autoimmune diseases, certain medications, and organ transplantation [1,3]. Emerging evidence suggests that substance use, particularly synthetic psychoactive drugs, may act as a trigger for aHUS in individuals who are genetically predisposed to complement dysregulation, rather than being an independent cause of the disease. A case report documented a patient with multiple complement mutations who developed aHUS after abusing synthetic psychoactive drugs, underscoring how external factors can aggravate underlying complement dysregulation [1]. This supports the “two-hit” hypothesis, where a genetic mutation and a secondary trigger are required to initiate aHUS, explaining the variability in clinical presentation [3].

Clinically, aHUS presents a broad spectrum of severity, from mild symptoms to rapid progression to multi-organ failure if not promptly recognized and treated. The disease mainly affects the kidneys but can also involve the central nervous, cardiovascular, pulmonary, gastrointestinal, and skeletal systems in up to 20% of cases, contributing to its high morbidity and mortality [2]. Distinguishing aHUS from other TMAs, such as thrombotic thrombocytopenic purpura (TTP) and STEC-HUS, is challenging due to overlapping features. A thorough diagnostic approach combining clinical evaluation, laboratory tests (e.g., hemoglobin levels, platelet counts, and lactate dehydrogenase), and genetic analysis is essential to ensure accurate diagnosis and guide appropriate management [2,3].

The management of aHUS has been significantly improved using complement inhibitors like eculizumab and ravulizumab. Eculizumab, a monoclonal antibody targeting the C5 component of the complement cascade, prevents the formation of the terminal complement complex (C5b-9), effectively controlling disease activity and reducing relapse rates [3]. Early administration of eculizumab is associated with better renal recovery and survival outcomes, especially in patients presenting with severe kidney involvement. However, the challenges of determining the optimal duration of therapy, managing relapses, and addressing the high costs associated with long-term treatment persist [3].

While current therapies have advanced the management of aHUS, there remains a need to further understand how various triggers, including infections, medications, and illicit drugs, contribute to the onset and progression of aHUS in genetically susceptible individuals. Filling these gaps is crucial for refining diagnostic protocols and developing more targeted therapeutic approaches considering genetic and environmental factors.

This narrative review aims to provide an updated synthesis of current evidence on aHUS, with a particular focus on its pathophysiological mechanisms, clinical presentations, and management strategies in the context of substance use as an environmental trigger. Additionally, the review highlights the interaction between genetic predispositions and substance-induced complement dysregulation, identifying areas for future research to better understand this relationship.

## Review

Methodology

A comprehensive literature search was conducted using three major databases: PubMed, Scopus, and Google Scholar. The search strategy was tailored to capture studies on substance-induced aHUS. Keywords such as “substance-induced atypical hemolytic uremic syndrome,” “drug-induced aHUS,” and “toxicological HUS” were used, combined with Boolean operators (AND, OR), to refine and broaden the search results. To ensure that only high-quality and relevant studies were included, specific inclusion and exclusion criteria were applied, as outlined in Table 1 below.

**Table 1:** Inclusion and exclusion criteria for selecting studies on substance-induced atypical hemolytic uremic syndrome (aHUS). HUS: hemolytic uremic syndrome; STEC: Shiga toxin-producing *Escherichia coli*.

Criteria	Inclusion	Exclusion
Language	Articles published in English	Articles not available in English
Type of studies	Peer-reviewed original research, including case reports, cohort studies, and clinical trials published after 2014	Non-original research (e.g., reviews, editorials, commentaries, and conference abstracts) and any paper published before 2014
Topic focus	Studies specifically addressing substance-induced atypical HUS (aHUS)	Studies focusing exclusively on typical HUS (e.g., STEC-HUS)
Outcome measures	Articles discussing the pathophysiology, diagnosis, management, or outcomes of substance-induced aHUS	Studies without a focus on substance-induced aHUS or unrelated to key review themes

The inclusion criteria focused on peer-reviewed articles published in English that provided original research on the etiology, clinical presentation, and treatment of substance-induced aHUS. In contrast, non-original articles, such as reviews, editorials, and commentaries, were excluded to ensure the synthesis was based on primary research data. Additionally, studies focusing solely on typical HUS (such as STEC-HUS) were excluded, as this review centers specifically on aHUS cases triggered by substance use.

The search process involved an initial screening of article titles and abstracts to assess their relevance. Full-text reviews were then conducted on articles that met the initial criteria to confirm their inclusion in the review. Multiple reviewers performed this selection process independently to minimize selection bias and enhance the reliability of the included studies.

Discussion

Pathophysiology

aHUS involves an impairment in the regulation of the complement cascade, triggered either by genetic predisposition or by spontaneous injury. This impairment results in excessive accumulation of complement proteins on endothelial cells, leading to the thickening of arterioles and capillaries and causing endothelial swelling and separation [4]. The activation of the complement system establishes a pro-thrombotic state, marked by the buildup of proteins and cellular infiltrates, including neutrophils, macrophages, and platelets, on the endothelial surface. These changes encourage the development of obstructive thrombi within blood vessels and mechanical fragmentation of red blood cells, forming schistocytes. This process leads to the classic triad observed in aHUS: Coombs-negative hemolytic anemia, renal impairment, and thrombocytopenia [5].

Microthrombi formation within the vasculature consumes platelets, leading to thrombocytopenia and obstructing blood flow. This obstruction shears red blood cells, contributing to hemolytic anemia. Renal dysfunction in aHUS is primarily attributed to the formation of microthrombi in the renal blood vessels. Anaphylatoxins, such as C3a and C5a, produced during complement activation, further exacerbate endothelial damage [5,6]. These inflammatory mediators worsen endothelial injury, creating a cycle of microvascular thrombosis. In substance-induced aHUS, illicit drugs such as heroin, cocaine, and synthetic psychoactive substances can act as significant external triggers. These substances may accelerate endothelial damage by promoting oxidative stress and inflammation, and enhancing complement activation, exacerbating complement dysregulation in genetically predisposed individuals [7]. Dysregulation of complement at the cell surface level is the primary mechanism driving aHUS, with approximately 20% of cases linked to familial factors [7,8].

Substance-induced aHUS emphasizes the role of environmental triggers in aggravating complement dysregulation. Genetic mutations, particularly in complement regulatory proteins such as CFH, CFI, CD46, C3, and CFB, are critical for disease susceptibility. When combined with exposure to illicit substances, the risk of complement overactivation increases significantly. This interaction between genetic predisposition and substance abuse leads to rapid disease progression and worsens endothelial injury [9]. Familial aHUS is diagnosed when at least two family members develop the disease within six months [9]. The genetic factors contributing to aHUS can present as autosomal dominant, autosomal recessive, single-gene pathogenic variants, or, rarely, as polygenic inheritance [10].

Mechanism of Injury

The excessive activation of the alternative complement pathway is the primary cause of injury in aHUS. This pathway is crucial in the immune system and is typically regulated by complement inhibitors to protect host tissues from damage. However, in aHUS, complement deposition on endothelial cells, thickening of arterioles and capillaries, and endothelial swelling and separation occur due to genetic factors or unidentified triggers that disrupt the complement cascade [11]. Some rare genetic variants of HUS, such as DGKE mutations and cobalamin C deficiency, do not affect the complement pathway directly [12,13]. Common triggers for aHUS include sepsis, viral infections, and pregnancy [14]. Regardless of the cause, aHUS is a rare disorder associated with poorer clinical outcomes and a higher risk of mortality and morbidity than infection-related HUS. Factors contributing to the over-activation of the alternative complement pathway in aHUS include genetic changes affecting complement proteins, such as FH, FI, FB, C3, and thrombomodulin, and the development of anti-FH autoantibodies [15].

In substance-induced aHUS, illicit drugs such as heroin, cocaine, and synthetic psychoactive substances can act as potent environmental triggers. These substances are believed to accelerate endothelial damage through oxidative stress and inflammation, further promoting complement dysregulation in genetically susceptible individuals. This leads to excessive complement activation, which compounds the existing genetic vulnerability by enhancing complement deposition on endothelial cells and amplifying the damage to the vascular system [16]. These substances intensify the activation of the membrane attack complex (MAC) (C5b-9), resulting in widespread endothelial injury and microvascular thrombosis [17].

During the acute phase of aHUS, granular deposits of C3 accumulate in the renal arterioles and glomeruli, triggering complement activation and local C3 consumption [16]. This effect can be significantly heightened in cases of substance use, where the inflammatory and oxidative responses to drug exposure exacerbate complement activation. Microvascular thrombosis in the kidneys occurs when the MAC (C5b-9) is formed [17]. The primary cause of aHUS is complement dysregulation at the endothelial cell surface, with familial factors accounting for approximately 20% of cases [8]. Although familial aHUS is less common than sporadic cases, understanding how genetic abnormalities affect the disease is vital for prognosis and treatment planning. Familial aHUS is defined as the occurrence of the disease in two or more family members within six months [10]. Genetic susceptibility to aHUS may manifest as autosomal dominant or recessive inheritance, typically involving mutations in genes that regulate the complement system, such as CFH, MCP (membrane cofactor protein or CD46), CFI, CFB, and C3, or, rarely, polygenic inheritance [18].

Diagnostics

The accurate diagnosis of aHUS, especially in contrast to other TMAs like STEC-HUS, is critical for appropriate management. STEC-HUS, diagnosed through stool culture or polymerase chain reaction (PCR), is approximately 10 times more prevalent than aHUS in Western countries [19]. Even when stool samples are negative for STEC, serological assays have identified recent STEC infections in numerous pediatric HUS cases. The diagnosis of aHUS relies on similar biochemical and hematologic criteria used for typical HUS and other TMAs. Atypical HUS should be considered in children under six months with a family history of HUS not presenting simultaneously, recurrent episodes, or the absence of bloody diarrhea. Gastrointestinal symptoms, particularly in children and adolescents with anti-FH antibodies, may indicate intestinal microangiopathic ischemia linked to aHUS [20].

The previous classifications of diarrhea-positive and diarrhea-negative HUS are now outdated. If diarrhea is brief or absent and HUS is present, aHUS should be considered over STEC-HUS, which typically appears four to five days after diarrhea onset [21]. In cases of substance-induced aHUS, diagnosing the condition can be even more challenging due to the unclear presentation of gastrointestinal symptoms and overlapping hematologic findings. In such cases, considering a history of drug use, including illicit drugs like heroin, cocaine, and synthetic psychoactive substances, becomes essential in evaluating possible environmental triggers for complement dysregulation. aHUS has a delayed onset with vague symptoms, reduced diarrhea frequency, low leukocytosis (below 16,000/μL), and increased coagulopathy [22]. Genetic polymorphisms enhancing complement function can cause aHUS even in cases involving STEC [23]. Anti-FH antibody-associated HUS was first described by Dragon-Durey et al. [20].

Pathogenic genetic changes and earlier gastrointestinal difficulties may delay the onset of aHUS [24]. Genetic variants affecting the CFH-related peptide and related processes have been documented in earlier studies. In cases of substance-induced aHUS, these genetic predispositions can be exacerbated by environmental factors, specifically drug exposure, making timely diagnostic workups critical. Distinguishing aHUS from TTP is essential for patients with TMAs. TTP is generally caused by congenital ADAMTS13 deficiency or the presence of anti-ADAMTS13 antibodies. ADAMTS13 plays a critical role in preventing the formation of von Willebrand factor multimers following platelet activation [25]. Thus, confirming ADAMTS13 activity is essential before diagnosing aHUS. The diagnostic approach should include ruling out STEC-HUS via stool tests, assessing ADAMTS13 activity to exclude TTP, and complement testing or genetic screening for aHUS. The diagnostic approach to substance-induced aHUS should involve a thorough investigation of the patient’s drug use history along with ruling out STEC-HUS via stool tests, assessing ADAMTS13 activity to exclude TTP, and complement testing or genetic screening for aHUS. Recent research has further advanced TMA diagnosis and management [26].

Treatment and Management

The management of aHUS, particularly in cases complicated by substance use, involves both supportive care and targeted therapies to address underlying complement dysregulation. Supportive management for acute kidney injury (AKI) focuses on preventing fluid overload, correcting electrolyte imbalances, controlling hypertension, discontinuing nephrotoxic medications, initiating dialysis when needed, and ensuring adequate nutrition. Blood transfusions are indicated for severe anemia (hemoglobin < 7 g/dL), but platelet transfusions should be avoided unless there is clinically significant bleeding, such as bleeding with hemodynamic instability, or a planned surgical procedure [27]. Plasma exchange and anticoagulation therapies have been explored since HUS was first described. The effectiveness of plasma therapy in aHUS is inconsistent and depends on factors such as complement factor deficiencies (CFH and CFI) or pathogenic antibodies (e.g., anti-FH antibodies) [27-29]. Plasma therapy, with or without exchange, remains a consideration but has limited effectiveness and concerns regarding volume overload, transmission of infections, and allergic reactions. These concerns are especially relevant in pediatric patients due to their smaller blood volume and higher risk of complications from catheter use [30].

Eculizumab (Soliris®, Alexion Pharmaceuticals, Inc., Boston, MA), a humanized monoclonal antibody against complement protein C5, has become a primary treatment for aHUS. By binding to C5, eculizumab prevents the formation of the terminal MAC (C5b-9), reducing endothelial injury. Eculizumab has demonstrated substantial efficacy in both pediatric [31-33] and adult [34] aHUS patients, leading to its FDA approval following several prospective studies, including one involving children [35]. Clinical trials have shown that eculizumab corrected blood lactate dehydrogenase levels, improved renal function, and normalized platelet counts in 82% of patients after 26 weeks, with 73% achieving a reduction in serum creatinine of 25% or more. Additionally, 82% of patients were able to discontinue plasma therapy without adverse outcomes like meningococcal infections or death. However, complications such as ischemic cardiomyopathy (reported in 2-4% of cases) and central nervous system issues (reported in up to 2% of cases) have been documented [34,36]. Eculizumab’s efficacy is generally not reliant on genetic mutations, as both patients with and without complement gene abnormalities can respond similarly, except in cases involving cobalamin C deficiency or DGKE mutations [37].

Ravulizumab, a next-generation complement inhibitor developed by Alexion Pharmaceuticals, Inc., was designed to address limitations associated with eculizumab, such as frequent dosing and related side effects. Modifications to the Fc region of eculizumab resulted in a four-fold longer half-life for ravulizumab, enabling extended dosing intervals through neonatal Fc receptor pathway recycling and prolonging terminal complement inhibition [38]. Approved for aHUS treatment in adults and children [27], ravulizumab has demonstrated comparable efficacy and safety to eculizumab in two 26-week phase III studies involving patients who were either naïve to complement inhibitors or previously treated with eculizumab. Ravulizumab was administered every four to eight weeks, and renal and hematologic parameters were maintained without significant adverse effects [39,40]. In treatment-naive patients, the most common side effects reported were headache, diarrhea, and vomiting. Although no direct comparisons exist between ravulizumab and eculizumab in aHUS patients, indirect comparisons using patient-level data from pivotal trials have shown no significant differences in platelet counts and renal outcomes at 26 weeks after adjusting for baseline characteristics [41]. The reduced dosing frequency with ravulizumab improves the quality of life for patients transitioning from eculizumab therapy [42,43]. Ravulizumab has been approved for treating aHUS in multiple countries [44].

**Figure 1:**
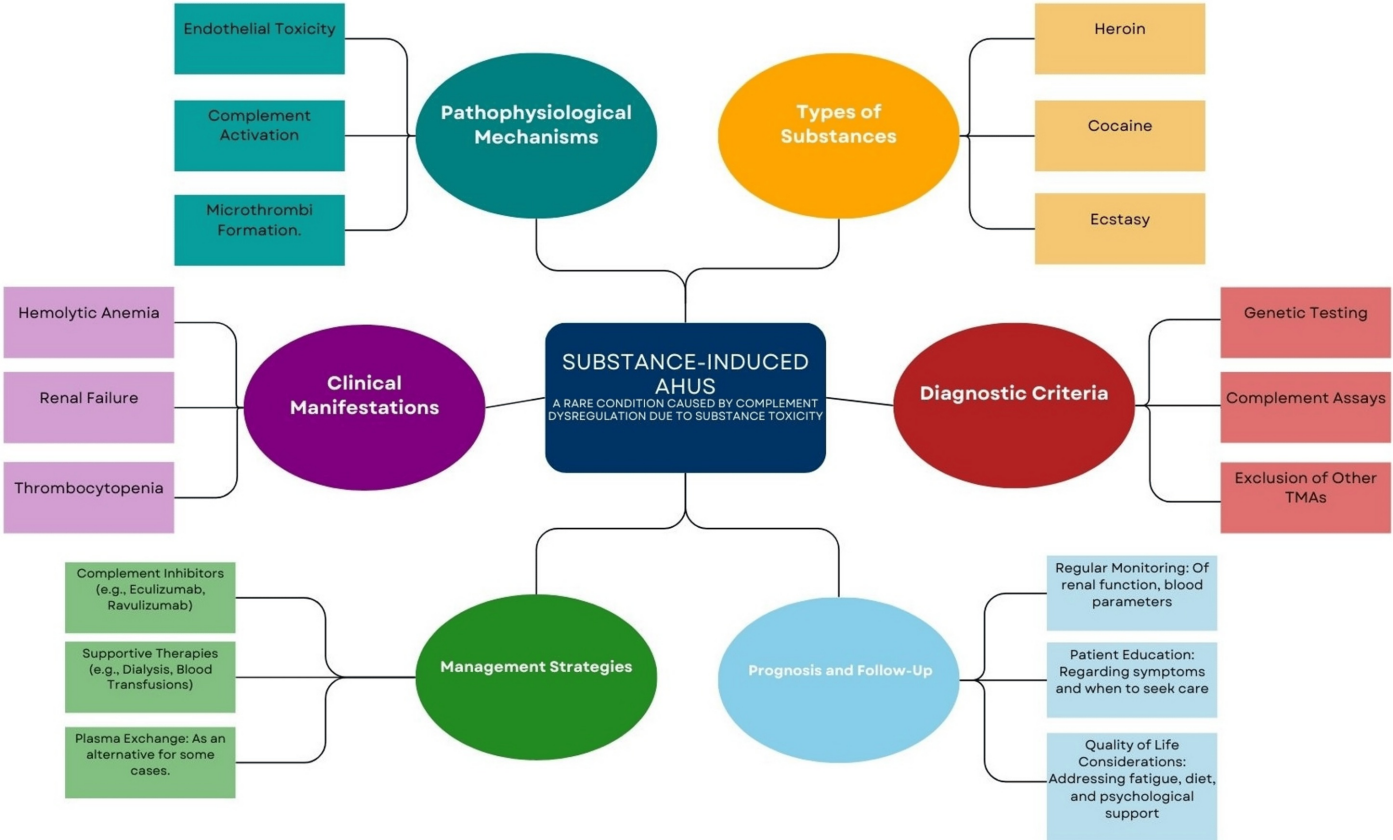
Overview of pathophysiology, diagnosis, and management of substance-induced aHUS. aHUS: atypical hemolytic uremic syndrome. Note: Figure 1 is the authors’ creation.

Figure 1 outlines the central elements involved in substance-induced aHUS, showing the progression from causative substances like heroin, cocaine, and ecstasy to key mechanisms such as complement activation. It illustrates how these pathways lead to clinical symptoms, including hemolytic anemia and renal failure, and guides the approach to diagnosis and treatment, emphasizing genetic testing and complement inhibitors. This visual representation provides a cohesive overview that supports the discussion on effective management strategies for substance-induced aHUS.

Summary of key studies

Research on substance-induced aHUS has investigated how various illicit drugs may trigger the condition in genetically predisposed individuals. Table 2 provides a concise overview of the most relevant studies, including the objectives, a summary of each study, and the authors' publication year. This table offers a snapshot of the existing literature, emphasizing the diversity of research conducted on substance-induced aHUS.

**Table 2:** Summary of key studies on substance-induced atypical hemolytic uremic syndrome (aHUS). CFH: complement factor H; CFI: complement factor I.

Sr. No.	Ref. No.	Author	Title	Year of publication	Objective to include in the study	Summary
1	[1]	Jelicic et al.	Atypical HUS with multiple complement system mutations triggered by synthetic psychoactive drug abuse: a case report	2023	Recent case report on substance-induced aHUS.	This case report emphasizes on the role of multiple complement mutations in the aHUS pathogenesis and highlights the importance of identifying genetic mutations in the complement system for accurate diagnosis and management of drug-induced aHUS.
2	[2]	Yan et al.	Epidemiology of atypical hemolytic uremic syndrome: a systematic literature review	2020	A systematic review covering aHUS epidemiology.	This review provides a comprehensive overview of the global epidemiology of aHUS, focusing on differences in disease prevalence, demographics, and outcomes, and explores the impact of various triggers, including drug abuse, in the onset of aHUS, underscoring the need for awareness in susceptible populations.
3	[3]	Raina et al.	Atypical hemolytic-uremic syndrome: an update on pathophysiology, diagnosis, and treatment	2019	Recent overview on aHUS, discussing pathophysiology and management.	This article discusses the role of complement dysregulation in aHUS, with a focus on how exogenous substances, like drugs, can exacerbate or trigger the condition. It also introduces refined diagnostic criteria for recognizing drug-induced aHUS.
4	[4]	Lebel et al.	Hemolytic uremic syndrome	2022	Recent, evidence-based review on HUS.	The authors summarize documented cases of aHUS triggered by various drugs and outline updated management protocols for patients with drug-induced aHUS.
5	[6]	Avdonin et al.	The role of the complement system in the pathogenesis of infectious forms of hemolytic uremic syndrome	2023	Discusses the role of the complement system, relevant to pathophysiology of aHUS.	Focuses on the mechanisms of complement activation in aHUS due to infectious agents and parallels with drugs and suggests potential therapies targeting the complement pathway applicable to drug-induced cases.
6	[7]	Fakhouri and Frémeaux-Bacchi	Thrombotic microangiopathy in aHUS and beyond: clinical clues from complement genetics	2021	Discusses genetic components and clinical clues for aHUS.	This article recommends routine genetic screening for at-risk patients, including those with drug exposure also elaborates on clinical features of drug-induced aHUS.
7	[12]	Donadelli et al.	HUS and TTP: traversing the disease and the age spectrum	2023	Recent discussion on the overlap between HUS and TTP.	The paper highlights the complex overlap between hemolytic uremic syndrome (HUS) and thrombotic thrombocytopenic purpura (TTP) across different age groups. It emphasizes that while both conditions share common features like microangiopathic hemolytic anemia and thrombocytopenia, they differ in underlying mechanisms, such as complement dysregulation in HUS and ADAMTS13 deficiency in TTP.
8	[13]	Furmańczyk-Zawiska et al.	Compound haplotype variants in CFH and CD46 genes determine clinical outcome of atypical hemolytic uremic syndrome (aHUS)—a series of cases from a single family	2021	Relevant to genetic aspects and clinical outcomes in aHUS.	It discusses how specific genetic haplotypes can influence the clinical outcomes of aHUS, relevant for cases involving drugs that may exacerbate these genetic risks, and advocates a personalized approach for the management of each case.
9	[14]	Spasiano et al.	Underlying genetics of aHUS: which connection with outcome and treatment discontinuation?	2023	Discusses genetics and outcome implications for aHUS.	It explains variations in complement regulatory genes, like CFH and CFI, that play a crucial role in determining clinical outcomes and guiding treatment discontinuation decisions in patients with atypical hemolytic uremic syndrome (aHUS). The authors provide specific criteria for discontinuing complement inhibitor therapy, balancing risks and benefits.
10	[16]	Yoshida and Nishi	The role of the complement system in kidney glomerular capillary thrombosis	2022	Relevant for understanding complement system involvement in aHUS.	Yoshida et al. (2022) identified specific biomarkers, such as elevated serum C5b-9 complexes and sC5b-9, that are indicative of complement activation linked to glomerular capillary thrombosis in atypical hemolytic uremic syndrome (aHUS). The study highlighted that exposure to certain drugs, such as calcineurin inhibitors, may exacerbate complement-mediated damage, contributing to glomerular capillary thrombosis.
11	[17]	Blasco et al.	Complement mediated endothelial damage in thrombotic microangiopathies	2022	Discusses complement-mediated damage, relevant to aHUS pathogenesis.	Blasco et al. (2022) describe that in drug-induced atypical hemolytic uremic syndrome (aHUS), endothelial damage is primarily driven by the dysregulation of the complement pathway, leading to the formation of membrane attack complexes (C5b-9) on the endothelial cells. This process results in direct endothelial injury, microvascular thrombosis, and the release of pro-inflammatory cytokines, further amplifying vascular damage and systemic inflammation.
12	[18]	Meyer et al.	Immunologic and genetic contributors to CD46-dependent immune dysregulation	2023	Discusses immunologic and genetic factors in aHUS.	This paper identifies mutations in the CD46 gene and autoantibodies against CD46 as key immunologic and genetic factors contributing to immune dysregulation in substance-induced atypical hemolytic uremic syndrome (aHUS). These alterations lead to impaired complement regulation on cell surfaces, increasing susceptibility to endothelial damage and microvascular thrombosis following drug exposure.
13	[24]	Raina et al.	Anti-factor H antibody and its role in atypical hemolytic uremic syndrome	2022	Discusses specific antibodies relevant to aHUS.	The article discusses the role of anti-factor H antibodies in the pathogenesis of aHUS, especially in cases linked to drug-induced immune dysregulation. Implications for therapy: Recommends treatments, such as plasmapheresis and complement inhibitors, for managing antibody-related cases.
14	[26]	Loirat et al.	An international consensus approach to the management of atypical hemolytic uremic syndrome in children	2016	International consensus on aHUS management.	This article presents consensus clinical practice recommendations generated by an international expert group of clinicians and discusses the importance of anti-factor H antibody, complement investigations, and continuation or cessation of anti-complement therapy in patients with and without identified complement mutations.
15	[27]	Gurevich and Landau	Pharmacological management of atypical hemolytic uremic syndrome in pediatric patients: current and future	2023	Discusses current and future treatments for pediatric aHUS.	The authors summarize that recent data favor the restrictive use of eculizumab in carefully selected atypical hemolytic uremic syndrome cases, but close monitoring for relapse after drug discontinuation is emphasized. Long-acting C5 monoclonal antibody ravulizumab enables a reduction in the dosing frequency and improving the quality of life in patients with atypical hemolytic uremic syndrome.
16	[28]	Sepúlveda et al.	Clinical presentation and management of atypical hemolytic uremic syndrome in Latin America: a narrative review of the literature	2024	Recent review on aHUS management in a specific region.	The review was conducted for a specific population, i.e., Latin Americans, and compared epidemiological, clinical, and genetic characteristics of aHUS with the rest of the world, it concludes that eculizumab substantially improved aHUS-related outcomes in almost all adult and pediatric patients.
17	[29]	Wei et al.	Application of eculizumab, a terminal complement inhibitor, in the management of atypical hemolytic uremic syndrome in a 14-month-old Chinese pediatric patient: a case report	2024	Case report on eculizumab use in aHUS management.	Presents a case of successful eculizumab use in managing a pediatric patient with drug-induced aHUS, demonstrating the drug's safety and efficacy. Also underscores variability in clinical presentation and a lack of standardized guidelines tailored to children, necessitating a more individualized therapeutic approach.
18	[41]	Tomazos et al.	Comparative efficacy of ravulizumab and eculizumab in the treatment of atypical hemolytic uremic syndrome: an indirect comparison using clinical trial data	2022	Comparison of key drugs used in aHUS treatment.	The study found that ravulizumab, with its extended dosing interval, provides comparable efficacy to eculizumab in managing drug-induced aHUS, while offering a reduced treatment burden due to less frequent administration. It also suggested that ravulizumab may result in similar clinical outcomes, such as improvement in kidney function and reduced hemolysis rates, with a more favorable safety profile.
19	[44]	Syed	Ravulizumab: a review in atypical haemolytic uraemic syndrome	2021	Review on the drug used in aHUS management.	The review emphasizes that ravulizumab, due to its long-acting C5 inhibition, offers a sustained therapeutic effect in patients with atypical hemolytic uremic syndrome (aHUS), reducing the need for frequent dosing while maintaining efficacy and safety comparable to eculizumab.

Limitations

This narrative review is subject to several limitations inherent to its methodology and the nature of the topic. Firstly, the narrative review approach is inherently prone to selection bias, as the inclusion of articles and studies is based on availability and relevance as perceived by the reviewers rather than a systematic, reproducible process. This introduces the risk of omitting relevant studies or including those that may not comprehensively represent the broader context of the subject.

The availability and quality of the existing literature also influence the findings. Due to the rarity of aHUS induced by substance abuse, much of the current evidence is derived from isolated case reports and small case series. These sources lack the robustness of more extensive observational studies or randomized controlled trials, limiting the ability to generalize the conclusions to all patients with substance-induced aHUS. Therefore, while the review offers valuable insights, the evidence base is inherently limited in scope and scale.

Additionally, no formal scoring system was employed to assess the quality of the included studies, which is a limitation of the narrative review approach. While this qualitative evaluation allows for a broader exploration of the topic, it lacks the rigor of systematic reviews or meta-analyses that utilize formal methodologies to assess study quality and minimize bias. Consequently, the strength and reliability of the evidence presented in this review may vary considerably between different studies, affecting the overall confidence in the conclusions drawn.

Finally, the scarcity of data on aHUS secondary to substance abuse remains a significant challenge, limiting the available evidence and diversity of reported cases. This lack of data can lead to an incomplete understanding of the full spectrum of clinical presentations, underlying pathophysiology, and treatment outcomes associated with this condition. As a result, establishing clear clinical guidelines or reliably predicting patient outcomes remains difficult.

## Conclusions

Substance-induced aHUS is a rare but severe disorder marked by complement system dysregulation, resulting in hemolytic anemia, renal failure, and thrombocytopenia. Proper differentiation from other TMAs, such as TTP and STEC-HUS, is crucial and requires a combination of clinical and genetic evaluations. The advent of complement inhibitors, including eculizumab and ravulizumab, has significantly improved patient outcomes by targeting the underlying pathophysiology, reducing the need for plasma exchange, and lowering relapse rates.

Despite these therapeutic advancements, challenges remain in optimizing treatment regimens and addressing risks such as infections linked to complement inhibition. This review highlights the need for more precise diagnostic criteria and personalized therapeutic approaches, particularly in substance-induced aHUS cases, where early diagnosis is critical to preventing irreversible organ damage. By focusing on the intersection of complement dysregulation and substance use, this work emphasizes the importance of individualized treatment strategies to improve long-term outcomes. Further research is essential to refine diagnostic protocols and develop personalized therapeutic approaches, especially for substance-induced aHUS cases.
